# Prevalence of Giardiasis and Entamoeba Species in Two of the Six Governorates of Kuwait

**DOI:** 10.1155/2022/5972769

**Published:** 2022-03-04

**Authors:** Rania M. AlAyyar, Abdullah A. AlAqeel, Muna Sh. AlAwadhi

**Affiliations:** ^1^General Sciences Department, Institute of Nursing, Public Authority of Applied Education and Training (PAAET), Shuwaikh, Kuwait; ^2^Laboratory Department, Alnafisi Dialysis Center, Ministry of Health, Shuwaikh, Kuwait

## Abstract

Intestinal parasitic infections are a global concern owing to elevated rates of morbidity and mortality in many parts of the world. Increased rates of intestinal parasitic infections are observed in developing and low-income countries. In Kuwait, many expatriates and foreigners hail from endemic countries, thus increasing the rate and risk factor of infection. This retrospective study is aimed at assessing the prevalence of *Giardia lamblia* and *Entamoeba* sp. in stool samples handled by two of Kuwait's general hospitals during the period from January 2018 to July 2019: Mubarak Al Kabeer (serving Hawalli governorate population) and Aladan hospitals (serving Mubarak Al Kabeer governorate population) serving 27% of total Kuwait population with Kuwaitis making up only 32%. A total of 9,653 samples were tested for consistency and the availability of any parasitic particles using visual, direct wet mount, and concentration method. Statistical analysis was implemented using SPSS statistical program, at *p* < 0.05. Of all the 9,653 tested stool samples, 74 were positive for *G. lamblia* or *Entamoeba* sp. This represented a mere 1% rate of infection and showed no significant correlation to the prevalence of intestinal parasite infections in Kuwait. On the other hand, comparing the rate of infection in different seasons revealed an increased rate of infection during spring compared to other seasons (*p* = 0.03). Findings revealed low rates of intestinal parasitic infection within the two governorates of Kuwait representing no statistical significance to the distribution of the investigated parasites in Kuwait. This may be attributed to improved living conditions and healthcare. Elevated infection rates in spring in relation to other seasons raised the importance of increasing public awareness during camping season for maintaining proper personal hygiene and waste management to contain and decrease the rate on infection.

## 1. Introduction

Intestinal parasitic infections (IPIs) are found to be one of the major public health problems worldwide [1–4]. An estimation by the World Health Organization (WHO) stated that over three billion people are infected with IPI worldwide, yet only 450 million are symptomatic [5]. Two of the most common IPIs are giardiasis and amoebiasis, where their mode of infection is highly dependent on the fecal-oral rout [6, 7]. This mode of infection may be acquired by direct or indirect transmission whether by person-to-person or indirectly through the use of contaminated food or objects [8, 9]. Giardiasis and amoebiasis are two of the most prevailing IPIs, arising from ingesting the infective cyst stage of the responsible parasite, needless of an intermediate vector [10–12]. Giardiasis, referred to as lambliasis as well, is caused by the parasite *Giardia lamblia* (also known as *G. duodenalis* and *G. intestinalis*), while amoebiasis is caused by *Entamoeba histolytica* [12–15]. Postingestion of giardiasis infective stage, cysts excyst in the duodenum releasing trophozoite form that will adhere to the surface of the small intestine microvilli, thus obstructing absorption function of the small intestine, or may move freely within the lumen [16, 17]. Though giardiasis is mostly asymptomatic, symptoms may prevail within 3 to 15 days postingestion, manifesting as a variety of symptoms, ranging from mild nutrient malabsorption and intestinal uneasiness up to a more acute stages with bloody, fatty stool, explosive diarrhea, vomiting, and anorexia [16–19]. With diarrhea being the second leading cause of morbidity and mortality due to infectious disease, proper diagnosis and treatment are essential [20]. In amoebiasis, the ingested infective cyst stage excysts in the small intestine as well but colonizes and invades the large intestine mucosal layer. Infection may develop further into an extraintestinal infection migrating to different parts of the host body including liver, brain, and lungs [21–23]. It is essential to note here that there are four morphologically identical *Entamoeba* species indistinguishable under the microscope, *E. histolytica*, *E. dispar*, *E. bangladishi*, and *E. moshkovskii*, with *E. histolytica* being the only causative agent of amoebiasis, while the latter three are considered commensal, noninfective species. Although recent studies have implicated the role of *E. dispar* and *E. moshkovskii* as potential pathogens to humans, yet a more precise diagnosis approach is essential in identifying the *Entamoeba* sp. under investigation to prevent unnecessary treatment [22–24]. According to the WHO guidelines, polymer chain reaction (PCR) is the approved method for identifying and differentiating the *Entamoeba* sp. [24]. Similar to giardiasis, almost 90% of amoebiasis infections are asymptomatic, while if symptomatic, infection may manifest in a range of symptoms from mild diarrhea to abdominal cramps, bloody, watery diarrhea, dysentery, and abscess formation [21, 22]. Both IPIs are found responsible for increasing morbidity in many parts of the world, especially in developing and low-income countries [5, 25, 26]. Amoebiasis is estimated to infect 480 million people globally, with elevated annual deaths ranging between 40 and over 100 thousand people, and nonetheless, giardiasis, while not life threatening, has an annual infection estimated at 280 million people worldwide [27–30]. According to the Global Burden of Disease Study 2010, the median global burden of giardiasis was estimated at 0.17 million, while the burden of amoebiasis was 0.5 million disability-adjusted life years [6, 31]. Some of the major risk factors involved in facilitating the spread of IPI are improper personal hygiene, feeble sanitary conditions, and deprived access to clean water supply [26, 32–34].

**Figure 1:**
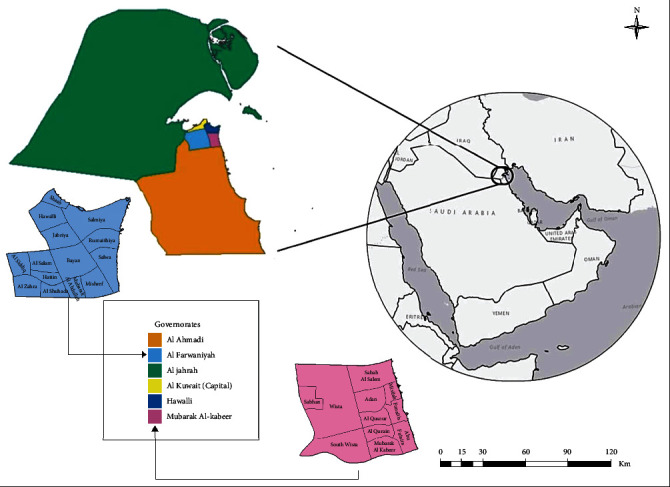
Map of Kuwait showing the six governorates and the cities of Hawalli and Mubarak Al Kabeer governorates [36, 40].

Kuwait is a developed country in the Middle East, positioned at the northeastern corner of the Arabian Gulf (Figure 1) [35, 36]. Kuwait attracts various employment seekers from all over the world, many of which hail from low-income or developing countries where elevated rates of IPI are recorded [2, 33, 37]. House staff, food handlers, healthcare personal, and many other occupations are considered high-risk factors for IPI transmission due to occupational nature, thus increasing infection exposure rates either by direct or indirect contact. According to the statistical reports of Kuwait's Public Authority of Civil Information (PACI) in 2019, total Kuwait population was stated to be 4,776,407, with Kuwaitis making up only 30% [38].

Several studies indicated the role of human mobility in the transmission of infectious diseases [39]. Shedding a light on the degree of IPI distribution in Kuwait could help in containing the infection. Hence, this study is aimed at estimating the prevalence of giardiasis and amoebiasis in two of Kuwait's governorates.

## 2. Materials and Methods

### 2.1. Study Site and Population

A retrospective study conducted on the archival records in two of Kuwait's public hospitals: Mubarak Al Kabeer hospital, which serves 16 cities comprising Hawalli governorate, and Aladan hospital, serving 13 cities comprising Mubarak Al Kabeer governorate (Figure 1). Both hospitals serve a total of 1,275,407 people, in which only 32% are of Kuwaiti nationality [38].

Hospitals' archival departments were the source of our data, retaining the confidentiality of all patient personal information and records. Ethical approvals and documentations were obtained from Kuwait Ministry of Health prior to data collection. Findings of all stool samples received by the microbiology laboratory department in both hospitals during the period from January 2018 to July 2019 were recorded. A total of 9,653 stool samples were handled by the microbiology laboratory department in both hospitals, 6,264 in Aladan hospital and 3,299 samples in Mubarak Al Kabeer hospital.

### 2.2. Sample Collection and Processing

Sample collection and processing in Kuwait hospitals and laboratories fall under the guidelines set by the Kuwait Ministry of Health in accordance with the WHO guidelines [41]. Stool samples are collected in a clean, wide-mouthed, plastic container with a tightly sealed cover. Upon receiving the samples, laboratory technicians check and record the sample consistency and presence of any mucus, blood, or parasitic materials and apply further preparation for microscopic examination using direct wet mount. Upon request or if sample seems soft or watery, concentration method is performed, using Fecal Parasite Concentrator (FPC) kit by Evergreen Scientific company. For wet mount method, a drop of normal saline is placed on one-half of a glass slide, and Lugol's iodine solution on the other half. A tiny portion of the stool sample is then applied to each solution and mixed well in preparation for microscopic examination. Concentration method, performed when required, follows similar guidelines applied in wet mount microscopic examination, yet the main solution used is normal saline, followed by another staining solution specific for the suspected form.

### 2.3. Statistical Analysis

Chi-square test was implemented to determine any correlation significance with the aid of SPSS version 25 (IBM, Franklin D Roosevelt, Manhattan, NY), and the *p* value considered significant at values <0.05.

## 3. Results

A total of 9,563 stool samples were processed by the microbiology laboratory department of both Aladan and Mubarak Al Kabeer hospitals. Recorded results for each sample were collected and classified according to the prevalence of *G. lamblia* and *E. histolytica/dispar* in each hospital and were further categorized according to season during which samples were collected in the period from January 2018 until July 2019.

From the 6,264 samples processed by Aladan hospital, 51 (0.81%) came positive, 43 with *G. lamblia* and 8 with *Entamoeba* sp. On the other hand, of the 3,299 samples processed by Mubarak Al Kabeer hospital, only 23 (0.70%) were positive for *Entamoeba* sp., lacking the presence of *G. lamblia*. Overall, a total of 74 positive samples were detected, 31 *Entamoeba* sp. (8 in Aladan and 23 in Mubarak Al Kabeer hospital) and 43 G*. lamblia* only in Aladan hospital representing only 0.77% of the 9,563 samples tested in both hospitals (Table 1).

Data collected was also classified according to season (Table 2). Highest rates of infection were observed in spring, with 35 positive samples, 16 G*. lamblia* detected only in Aladan hospital and 19 *Entamoeba* sp., 6 in Aladan and 13 in Mubarak Al Kabeer hospital, making up 1.19% of the total samples received during the spring season. These findings represented 47% of total infected samples in our study. Significant correlation was observed between the rate of infection and different seasons with the *p* value estimated at 0.033.

## 4. Discussion

It is well established that IPI is a worldwide burden, owing to increasing morbidity and mortality [42–45]. According to the WHO, over two billion people are currently harboring IPI throughout the world [46]. Poor deprived communities with low socioeconomical and educational status, where improper waste and sewage management, poor hygienic habits, limited access to clean water supply, and ignorance of appropriate personal practices are more prone to IPI dissemination [42, 45, 47–51]. In Kuwait and other Arabian Gulf countries, many expatriates and residents originate from these parts of the world thus implementing a great risk factor to the spread of intestinal parasites due to occupational nature [1, 5, 51]. Previous studies within Arabian Gulf countries estimated the prevalence of IPI to range between 2.3 and 47%, 7.7% in the UAE, 10.2% in Qatar, and 2.3-47% throughout the KSA [47, 51]. Our study focuses on the prevalence of giardiasis and amoebiasis in only two of Kuwait's highly populated governorates, Hawalli and Mubarak Al Kabeer. Of the 9,563 stool samples tested, only 74 were recognized with parasitic findings. This represented 0.77% infection rate showing insignificant correlation to the spread of IPI in Kuwait, driving against our estimated hypothesis. This may be attributed to the improved lifestyle and living condition and proper healthcare in Kuwait or the fact that most samples belong citizens due to recently enforced medical fees refraining many foreigners from pursuing proper testing. The latter cannot be confirmed currently due to unavailability of personal data, nationality, on samples investigated in this study; thus, a more targeted approach is recommended emphasizing participants' details on a personal level.

Differentiating morphologically identical *Entamoeba* sp. was not established since the required method (i.e., PCR) is not routinely performed in the Kuwait Ministry of Health. Amoebic findings were thus generalized and expressed as *Entamoeba* sp. (*E. histolytica/E. dispar/E. bangladeshi/E. moshkovskii* complex).

Data was further analyzed according to season, revealing significant correlation, at *p* = 0.033, to spring over other seasons which was consistent with previous reports [47]. Most common spring activity in Kuwait is desert camping, where limited access to running water and improper waste management are a main concern. Thus, public awareness and education on proper hygienic performances and waste management during camping season are essential to withhold against the spread of parasites.

## 5. Conclusion

Our finding revealed a low rate of giardiasis and amoebiasis in two of Kuwait's six governorates, representing insignificant risk factor for IPI. This may be attributed to enhanced living conditions, accessible healthcare throughout Kuwait, and the role of public awareness in limiting the spread of transmitted diseases on a global scale.

During camping season, infection rates were assessed highest, emphasizing the importance of public education and awareness on proper personal hygienic approaches and waste management to ensure withholding the spread of parasites.

## Figures and Tables

**Table 1:** Prevalence of parasitic infection in Hawalli governorate (Mubarak Al Kabeer hospital) and in Mubarak Al Kabeer governorate (Aladan hospital).

Hospital	Total samples	Infected	(%)	*G. lamblia*	*Entamoeba sp.*
Aladan	6,264	51	0.81%	43	8
Mubarak Al Kabeer	3,299	23	0.70%	Null	23
Total	9,563	74	0.77%	43	31

**Table 2:** Prevalence of *G. lamblia* and *Entamoeba* sp. in different seasons (2018-2019).

Parasite	Winter		Spring		Summer		Autumn	
	No.	*p*(%)	No.	*p*(%)	No.	*p*(%)	No.	*p*(%)
*G. lamblia*	12	0.48%	16	0.54%	13	0.35%	2	0.43%
*Entamoeba* sp.	4	0.16%	19	0.65%	10	0.27%	0	0.00%
Total positive (%)	16	0.64%	35	1.19%	23	0.63%	2	0.43%
Total samples	2487		2944		3663		469	

## Data Availability

Data used to support this study are fully available, and detailed data are available from the corresponding author upon request.
